# Bridging the Gap from Student to Doctor: Developing Coaches for the Transition to Residency

**DOI:** 10.1080/10872981.2022.2145103

**Published:** 2022-11-09

**Authors:** Abigail Ford Winkel, Colleen Gillespie, Agnes Park, Jeremy Branzetti, Patrick Cocks, Richard E. Greene, Sondra Zabar, Marc Triola

**Affiliations:** a Department of Obstetrics and Gynecology, Assistant Director for Education Scholarship in the Institute of Innovations in Medical Education and Co-Director of the Transition to Residency Course at the NYU Grossman School of Medicine, New York; b Department of Medicine and Director of the Division of Education Quality at the NYU, Grossman School of Medicine; c Department of Medicine at the NYU, Grossman School of Medicine; d Emergency Medicine Physician at Geisinger Community Medical Center, Geinsinger Health System, Danville, PA; e Department of Medicine and the Director of the Internal Medicine Residency Program at the NYU, Grossman School of Medicine; f Department of Medicine and the Director of the Division of Education Quality at the NYU, Grossman School of Medicine; g Department of Medicine, and Director of the Standardized Patient Program at the NYU Grossman School of Medicine; h Department of Medicine and Associate Dean for Educational Informatics and Director, Institute for Innovations in Medical Education at NYU Grossman School of Medicine

**Keywords:** Coaching, transition to residency, faculty development, graduate medical education, master adaptive learner

## Abstract

**Background:**

A lack of educational continuity creates disorienting friction at the onset of residency. Few programs have harnessed the benefits of coaching, which can facilitate self-directed learning, competency development, and professional identity formation, to help ease this transition.

**Objective:**

To describe the process of training faculty Bridge Coaches for the Transition to Residency Advantage (TRA) program for interns.

**Methods:**

Nineteen graduate faculty educators participated in a coaching training course with formative skills assessment as part of a faculty development program starting in January 2020. Surveys (n = 15; 79%) and a focus group (n = 7; 37%) were conducted to explore the perceived impact of the training course on coaching skills, perceptions of coaching, and further program needs during the pilot year of the TRA program.

**Results:**

Faculty had strong skills around establishing trust, authentic listening, and supporting goal-setting. They required more practice around guiding self-discovery and following a coachee-led agenda. Faculty found the training course to be helpful for developing coaching skills. Faculty embraced their new roles as coaches and appreciated having a community of practice with other coaches. Suggestions for improvement included more opportunities to practice and receive feedback on skills and additional structures to further support TRA program encounters with coaches.

**Conclusions:**

The faculty development program was feasible and had good acceptance among participants. Faculty were well-suited to serve as coaches and valued the coaching mindset. Adequate skills reinforcement and program structure were identified as needs to facilitate a coaching program in graduate medical education.

## Main Text Introduction

A lack of developmental continuity between undergraduate (UME) and graduate medical education (GME) creates disorienting friction at the onset of residency training [1,2]. An abrupt transition results in lost momentum in professional growth, as well as problems with wellbeing and concerns about the quality and safety of patient care [1,2]. By contrast, an optimized transition to residency should foster new doctors who are motivated – and not overwhelmed – by novel challenges as they grow their nascent professional identities [3].

Developmental trajectories in GME are unique, based on the strengths, weaknesses, and clinical experiences of each physician, as well as the sociocultural context of their learning environment. A one-size-fits-all approach is inadequate to address the individual needs of learners [4]. Yet, most programs to ease the transition to residency fall short of embracing a learner-centric approach, and traditional academic relationships in GME (e.g., mentor-mentee, advisor-advisee) are set up such that learners are offered advice or direct solutions to their problems [4,5]. New structures for supervision are needed to provide educators with the skills and opportunities to guide learners towards independent reflective thinking and analysis [3].

Over the past decade, coaching has been identified as a means to facilitate self-directed and individualized learning, competency development, and professional identity formation in medical students – all attributes that would also help interns better navigate the transition to residency [5–8]. Unlike traditional approaches for working with learners as advisors or mentors, where the expertise of the faculty member influences guidance given to learners, coaching is a unique paradigm that ultimately places emphasis on the learner as the driver of their own knowledge [8]. Educators use exploratory questions to help learners identify their goals and action plans, and positively reframe current issues in their development trajectory as stops along the road to a better future [8]. Coaching can facilitate the development of mastery learning behaviors by encouraging learners to engage in continuous learning cycles and deliberate practice [9–11].

To date, few residency transition programs have harnessed the benefits of coaching to address the needs of graduating students and interns [12,13]. Furthermore, there is limited data around the experiences of faculty coaches as they switch over to a coaching role from more traditional roles in GME [14]. The aim of this work is to describe our experiences developing faculty educator coaches for a UME to GME transition program. The question this research aims to answer is how medical education faculty experience coaching training, and what is needed to support effective coaching for learners in graduate medical education. We report on their performance of coaching skills during a formative group assessment and explore their experiences and perceptions of coaching during the pilot year of a transition-to-residency coaching program.

## Materials and Methods

In 2019, clinician-educators and medical education researchers at the New York University Grossman School of Medicine (NYUGSoM) received a five-year grant through the American Medical Association’s (AMA) Reimagining Residency Initiative to establish the Transition to Residency Advantage (TRA) coaching program. The TRA program was designed to match incoming residents from five collaborating residency programs (Internal Medicine, Obstetrics & Gynecology, Orthopedics, Pathology and Emergency Medicine) with a Bridge Coach from their department, whom they would work with throughout the first year of residency. Each program selected the coaches based on their individual faculty and structural considerations. Coaches filled a variety of other roles in education, including associate residency program director, transition to residency program director, resident mentor and clinical educator. A structured curriculum was developed, which defined a schedule for coaching and set the agenda for each coaching session.

Bridge Coaches were assigned to their first group of interns on Match Day 2020. Between the AMA grant and matching funds from the institution, coaches had 0.1 full-time equivalent (FTE) support for their coaching activities with the expectation that they would have roughly ten assigned coaches. The actual number of coaches per coach varied between residency programs (range = 1–18) depending on program factors, such as class size and the dedicated time faculty had for coaching. In programs where faculty had less dedicated time, incoming interns were assigned to a group of Bridge Coaches.

The study of this program is a mixed methods analysis of assessment data, surveys and focus groups with coaches. All methodologies used to evaluate the TRA program were approved by NYUGSoM’s Institutional Review Board (protocol i19-01065).

### Faculty development program for training Bridge Coaches

A faculty development program was created to provide coaching training to established UME and GME educators and develop them into TRA Bridge Coaches. In 19 January 2020 faculty participated in a synchronous, in-person coaching training course comprised of ten sessions (eight small-group workshops, one didactic lecture, one structured group evaluation). The curriculum covered coaching fundamentals, techniques and resources to foster learning and growth, and TRA program specifics (e.g., TRA app, structured activities). Emphasis was placed on learner-centeredness, comprehension of the specific needs and challenges of interns, and reflective thinking on how coaching can fit and be impactful within GME.

A Group Objective Structured Coaching Evaluation (GOSCE) was utilized for formative assessment of faculty’s coaching skills. Faculty engaged in group role play with standardized learners and participated in a debriefing session. Observational data were collected by peer and resident observers using a structured, behaviorally anchored grading rubric (see Table A1) designed to assess skills and provide feedback in four coaching domains: 1) establishing trust and rapport; 2) authentic listening; 3) asking questions; and 4) coachee-focused agenda. Possible ratings were ‘not done,’ ‘partly done,’ and ‘well done.’

Throughout the TRA program year, Bridge Coaches participated in monthly, hour-long virtual meetings led by the TRA program co-director for continuation of skills development and support of the TRA program. Coaches have the opportunity to review practical questions related to the program’s curriculum and activities and discuss coaching challenges with peers.

A detailed outline of faculty development program activities is provided in the Table A2.

### Focus Group and Survey with Bridge Coaches

In November 2020, a virtual focus group was conducted to learn about faculties’ coaching experiences thus far. The focus group was facilitated by one of the authors (CG), who was not directly involved in the curriculum, and lasted about one hour. Semi-structured questions were used to explore participant’ opinions and perceptions of coaching – particularly in regards to what felt novel or challenging – and how they had incorporated coaching into their interactions with learners. Audio from the focus group was recorded and sent to a third-party company for transcription and de-identification.

Between June and July 2021, coaches were asked to complete an online survey assaying the perceived impact of the faculty development program curriculum on core coaching skills identified from Wolff et al.’s five-domain competency framework for coaching in medical education.^12^ Along with session attendance, this data was utilized to assess feasibility and acceptance of the curriculum. Skills were assessed using Likert-type and open-ended survey questions in three competency domains: 1) Coaching Process and Structure; 2) Relational Skills; and 3) Coaching Skills.

The focus group transcript and written survey responses were independently reviewed by AFW and CG using simple content analysis. Main themes and subthemes were identified and differences between reviewers were discussed until consensus.

## Results

Participants attended an average of 8.7 out of 10 training sessions (SD 2.1) of the faculty development program. All Bridge Coaches attended 4 or more sessions, with roughly half attending all sessions and 35% attending 8 or 9 sessions. At the time of participation, all but one of the coaches were also working with residents as advisors, mentors, or supervisors.

Feedback provided by faculty in the focus group and written survey responses indicated good acceptance of the faculty development program by participants. The majority of statements participants made about the program reflected a positive experience of the coaching training course (‘I think it went really well’) and considered its content and activities to be valuable (‘They are excellent. Love the small group sessions. The role play was helpful.’). Participants greatly appreciated the monthly meetings with other Bridge Coaches and expressed wanting more opportunities to meet as a group to discuss challenges and share solutions between programs. Other suggestions for improvement included having more opportunities to repeat concepts and practice skills during and after the coaching training course, and adding more structure to scheduled meetings with coaches.

### Evaluation and feedback of faculty coaching skills

Participants’ performance on the formative GOSCE are presented in Table 1 as the proportion of resident and peer ratings that were ‘well done’ for the four assessed domains. The highest proportion was in *Authentic Listening* (74.1% of resident ratings, 72.2% of peer ratings), followed by *Asking Questions* (59.3% resident, 66.7% peer). Proportions for the domain *Establishing Trust and Rapport* were 61.1% and 58.3% for resident and peer ratings, respectively. Lastly, proportions for *Coachee-focused Agenda* were 45.4% and 58.3%, respectively.

**Table 1.** Ratings of faculty’s coaching skills given by standardized residents and peer observers during the Group Objective Coaching Skills Examination.

Coaching Domain	Specific Skills	Resident Ratings	Peer Ratings
Establishing Trust and Rapport*	Created a trusting environment	61.1%	58.3%
	Acknowledged strengths		
Authentic Listening	Listened without expressing opinions	74.1%	72.2%
Asking Questions	Explored emotional content of situations	59.3%	66.7%
	Summarized and mirrored without judgment		
Coachee-Focused Agenda	Supported resident in finding the solutions	45.4%	58.3%
	Future-focused goal-setting		
	Model willingness to learn and humility		

*All ratings based on % ‘well-done’ (see Appendix for Assessment Rubric) on a behaviorally anchored scale (see *Appendix* for assessment rubric).

### Perceived Impact of the coaching training curriculum on coaching-related skills

The online survey was completed by 15 (79%) Bridge Coaches. Prior to taking part in the faculty development program, 74% had no formal training in coaching and 68% had little to no experience providing or receiving coaching. Responses to the following question survey item are summarized in Table 2: ‘Please indicate the degree to which the [coaching training course] helped improve your skills in each area.’

**Table 2.** Faculty perceptions on the degree to which the coaching training program helped improve coaching skills in specified areas (provided on a 4-point Likert scale*).

Area/Skill	Mean	Std Dev
**Coaching – Process and Structure**	**2.90**	.**67**
Establishing the coaching agreement	3.17	.84
Coach self-monitoring	3.00	.60
Managing process and accountabillity	2.92	.79
Meeting management	2.50	.91
**Coaching – Relational Skills**	**3.00**	.**82**
Establishing meaningful coaching relationships	3.58	.66
Effective communication	3.00	.83
Adaptability	2.75	1.07
Emotional intelligence	2.67	1.01
**Coaching – Coaching Skills**	**3.10**	.**64**
Facilitating coachee well-being and professional fulfillment	3.42	.79
Stimulating others to develop own professional development learning objectives	3.33	.78
Helping others become aware of emotions that influence their own behavior	3.17	.72
Helping others investigate their behavior from a distance	3.08	.67
Helping others recognize personal feelings	2.83	.72
Fostering the development of master adaptive learners	2.75	.95

*1 = Curriculum Did NOT Improve My Skills At All. 2 = Curriculum Improved My Skills a Little. 3 = Curriculum SOMEWHAT Improved My Skills. 4 = Curriculum Improved My Skills Alot

Overall, participants reported improvement in coaching skills between ‘somewhat’ and ‘a lot.’ Mean participant scores for the three coaching domains were 3.1(0.6) for *Coaching Skills*, 3.(0.8) for *Relational Skills*, and 2.9(0.7) for *Process and Structure*. The skill with the greatest perceived change in all domains was *establishing meaningful coaching relationships*, which had a mean score of 3.58[0.66]. This was followed by *facilitating coachee well-being and professional fulfillment* (mean = 3.4[0.8]) and *stimulating others to develop their own professional development learning objectives* (mean = 3.3[0.8]), both in the *Coaching Skills* domain. The curriculum helped participants improve ‘a little’ to ‘somewhat’ in six skills. Least improvement was reported for *meeting management* (mean = 2.5[0.9]) and *fostering the development of master adaptive learners* (mean = 2.8[0.95]).

### Faculty perceptions of coaching in GME

Seven (37%) GME Bridge Coaches participated in the focus group. Data from the focus group and responses to open-ended survey questions revealed three major themes related to participants’ experiences and perceptions of coaching:

- Adopting a coaching perspective represents a paradigm shift

Engaging in a coaching relationship with residents was a distinctly new experience for Bridge Coaches. Coaching required participants to use a different mindset, approach, and skillset than what they were accustomed to from their non-coaching faculty roles. They found that using coaching skills empowered their learners in a way that felt personally fulfilling. Participants recognized the powerful motivation that learners could derive from self-discovery.

- Layering coaching onto existing educational roles

The participants held other positions as their coachees’ advisor/supervisor through their other educational roles. They noted challenges navigating two roles that were seemingly at odds with each other. However, participants appreciated having an insider perspective while coaching and felt they were able to utilize coaching in a potentially more interesting and engaging way for residents.

- Coaching faculty development and the community of practice

Participants comments reflected that they viewed themselves as a community of practice. They were driven to work collaboratively to improve their coaching skills. Participants greatly valued the insight and perspectives of the other coaches.

Subthemes and exemplar quotes from the focus group and survey responses are provided in Table 3.

**Table 3.** Identified themes during qualitative analysis of faculty reflections on coaching from focus group and open-ended survey responses

Major Themes and Sub-Themes	Exemplar Quotes
**Theme 1: Adopting a coaching perspective represents a paradigm shift**	
SUBTHEMES Transitioning to coaching	I’ve been much more intentional … asking that question to the resident of what they are working on, what are they thinking about, what are they concerned about; and being much more intentional in my listening. [Focus Group, Bridge Coach]
Supporting self-discovery/self-directed learning	Focusing on helping our mentees become more intentional about their approach to residency. Watching them push themselves through residency helping to keep their mindset on their learning, education, and growth has been very rewarding. [Survey Response]
Goal-setting	Our focus on learning how to help them come up with tangible and actionable goals has been particularly meaningful. [Survey Response]
Incorporating resilience/wellness	The well-being area is an area where I think I have been coaching before even coaching, because I feel like that’s got to come from the resident. It’s not something I can give advice on but I can certainly host a conversation and get them to reflect and set goals for sure. [Focus Group, Bridge Coach]
Resident engagement in coaching	Coaching success feels highly dependent on the engagement of the learner – some take it seriously, are thoughtful and share their vulnerable side, others not so much. [Survey Response]
**Theme 2: Layering Coaching onto Existing Educational Roles**	
Balancing coaching role with other roles	But I want to coach, and I kind of want to coach the people who I get to know really well through my advisor relationship. If I’m getting to know them anyway, I really would rather get to know them as their coach. It makes the advisor role relationship a little stale. [Focus Group, Bridge Coach]
	I appreciate the tension between being a supervisor and a coach. We are coaches and supervisors because of efficiency. There’s no one else who will do it for free. We are adding it on to the other work that we’re doing. It’s sort of the dirty reality of the GME. [Focus Group, Bridge Coach]
**Theme 3: Coaching Faculty Development and Community of Practice**	
Learning from each other/co-creating coaching	Most helpful have been the group conversations and hearing the different perspectives everyone brings to coaching. Just talking about coaching and all the different techniques and styles has helped me become more intentional and thoughtful. [Survey Response]
Skills practice and reinforcement	I would want someone to be in there with me and be like, “there was a coaching opportunity that you could have taken that you just breezed right by or were really cavalier about or whatever.” [Focus Group, Bridge Coach]
New coaching role requires new structure	I love the sessions, and we talk about them after the sessions, and then they kind of go to the back of my mind…it would be helpful to have reminders or coaches or something that – I’m not sure the sessions are enough without having more clear goals for ourselves. [Focus Group, Bridge Coach]

## Discussion

As experienced mentors and advisors in medical education, the faculty that participated in our faculty development program already possessed many of the essential skills needed for coaching, such as the ability to build rapport with learners and support goal-setting practices. They required more practice with newer skills intended to facilitate the learner-driven approach of coaching – such as asking probing questions to guide learners toward self-discovery of solutions (rather than providing them) – and noted the novelty of taking on a coaching mindset as a GME educator. Insights provided by Bridge Coaches underscore the importance of having sufficient opportunities for practice and feedback to reinforce coaching skills, and the need for adequate structure to support a coaching curriculum in GME.

This is a novel use of coaching to bridge the educational continuum from the end medical school and into residency. Given the unique structures of GME, having faculty embrace the coaching role while occupying other roles within the residents’ clinical learning environment is an important factor to consider to bring coaching experiences close to the authentic learning of residency. The TRA faculty development program was feasible and had good acceptance among participants. Faculty found that using coaching skills motivated and empowered their learners in a way that felt personally fulfilling to themselves. They expanded their professional identities to embrace their new roles as coaches, aligning with previously reported findings in the literature [14]. By being intrinsically motivated to continue their professional development and working to change the learning culture within GME, they, in many ways, co-produced the goal of coaching.

It is not yet clear what structures for coaching enable optimal engagement with residents. There is an inherent tension between the structure needed in medical education and the learner self-directedness necessary for coaching. Unlike traditional dedicated coaching programs, faculty working in GME often have access to evaluation data, can work as direct supervisors, and may even be involved in promotion and remediation decisions (e.g., serve on the Clinical Competency Committee). Thus, creating safe spaces for residents to be open, reflective, and vulnerable in coaching remains an area of attention. Positively, prior coaching studies have shown that GME learners may uniquely benefit from having coaches who are also authentic role models that possess a deep understanding of the complex learning needs of residents [12,15]. Introducing coaching by way of established faculty may have the potential to infiltrate the culture of the learning environment, promote a growth mindset, and celebrate the continuous learning required of physicians to ensure the best care of their patients.

In light of the positive feedback to coaching from the initial group of faculty coaches, as well as interest from other faculty to receive training, an asynchronous model of the training curriculum was created to expand faculty development in coaching throughout our institution. Using video lessons, independent readings, exercises, and small-group work, this curriculum provides a more flexible approach to training educators who are not able to dedicate time to a scheduled workshop series. This curriculum is available online at: https://navigator.iime.cloud/course/coaching/.

The limitations of this project are its small scale and unique setting at an academic medical center where 30–40% of graduating medical students continue for residency each year. Data was collected to assess the experience of the program retrospectively, and not prospectively, which is a limitation of the design. Creating meaningful connections with learners prior to residency starting may be challenging in some settings. However, due to the limitations of the COVID-19 pandemic, the curriculum and coaching program took place in a largely virtual setting, which opened opportunities for expanding the reach of the curriculum. Increased comfort with virtual platforms facilitates the ongoing connection between coaches within the community of practice.

Research is currently underway to better understand what trainees’ experiences are with the TRA coaching program, and how their interactions with GME Bridge Coaches inform their attitudes, well-being, goal-setting, and performance. Future research must examine how integrating coaching changes learner trajectories and performance. We aim to learn whether integrating coaching within the constraints of GME can influence the learning culture positively and cultivate the growth mindset and self-directed learning necessary for master adaptive learners.

The promotion of learner agency, individual development, and resident well-being makes coaching well-suited to help ease the challenges of the UME to GME transition. Faculty were well-suited to serve as coaches for first-year residents, and felt fulfilled by their new roles. Continuous reinforcement of novel coaching skills and structures to support coaching encounters are necessary to facilitate coaching in GME.

## Appendix

**Table A1.** Group Objective Structured Coaching Evaluation Rating Rubric and Feedback Form.

Coaching Skills	Not Done	Partly Done	Well Done	Comments
***Establishing Rapport and Trust***				
Created a trusting environment	Did not address issues of confidentiality of coaching session	Stated that the coaching session was confidential without explaining what that meant or addressing nuances of relationship with faculty member serving as coach	Discussed the importance of confidential coaching discussions, and also acknowledged conflicts that might exist when coach might serve in evaluator, mentor or advisor roles as well as safety issues when relevant.	
Acknowledged strengths	Did not provide encouraging comments	Provided positive support that could be conceived as judgments of performance rather than identification of personal strengths	Acknowledged positive qualities (humor, eagerness to learn, courage) in the resident without focusing on specific tasks and achievements	
***Asking Questions***				
Asked useful questions	Validated or challenged statements without deepening the conversation to gather data	Asked a few but not many questions to guide reflection	Demonstrated curiosity and asked probing questions to encourage or challenge, gather more data	
Pacing questions	Asked questions that were too long and complex	Asked short questions but did not give adequate time for reflection and response	Asked questions in a manner that allowed the resident time to provide a response	
Prompting deeper insight	Did not provide scaffold for exploring issues or ask follow up questions	Asked follow up questions but did not provide tools or strategy to explore issues	Used questions to increase understanding of problem or provided tools to explore values and priorities as well as barriers (e.g., vision exercise, use of time-management matrix)	
***Authentic Listening***				
Listened without expressing opinions	Expressed approval, agreement, reproach or disapproval which directed the resident towards a specific response	Provided some comments that indicated the coach’s opinion of behavior but did not interfere with the resident’s narrative	Made neutral comments and asked neutral questions to facilitate complete expression of resident’s experience	
Explored emotional content of situations	Does not identify emotional content of situations the resident describes	May name the emotions the resident is feeling but does not encourage reflection on them	Prompted the resident to identify emotions as well as how that informs their reactions to a situation	
***Coachee-Focused Agenda***				
Summarized and mirrored without judgment	Derailed discussion from resident experience by sharing too much of the coach’s personal experience	Repeated but did not summarize or clarify the situation presented by the resident	Used resident’s own words to summarize situations and asked if the summary reflected the resident’s experience/feelings	
Supported resident in finding the solutions	Gives specific advice instead of asking questions to help resident arrive at insights	Stated it was the resident’s role to find the answers, but did not provide support	Encouraged resident to think creatively about potential solutions	
Future-focused goal-setting	Focuses on past experiences rather than future applications	Allows the resident to speak about future plans in a general, non-specific way	Helps resident to identify specific goals, options and next steps	
Model willingness to learn and humility	Did not ask for feedback about encounter or encourage 2-way dialogue	Asked for feedback in a general way (e.g., “how did this go for you?)	Encouraged a 2-way dialogue to ensure coaching meetings were optimally useful for resident or asked for specific feedback about what worked or didn’t in the encounter	

## Table A2.

**Table:** Faculty development program for Bridge Coaches.

	Session Title	Objectives/Activities
1	Introduction to Coaching in Medical Education (Workshop)	Differentiate between the role of coach, advisor and mentor, and describe how relate to each other in GME. Reflect on prior experiences of coaching and practice techniques using visioning exercise.
2	Fostering Trust Through Active Listening & Powerful Questions (Workshop)	Review strategies and language useful top optimize coaching Practice in triads of coach-coachee-observer and receive feedback. Discuss challenging cases from prior experience.
3	Group Objective Structured Coaching Evaluation (GOSCE)	Formative assessment of coaching skills
4	Strengths-Based Coaching (Workshop)	Participate in a self-assessment and reflection using an online strengths assessment. Consider how to use a perspective on strengths to guide feedback and behavior change.
5	Transitioning to Independent Learning in Residency (Lecture)	Describe the features of master adaptive learners, including understanding how processing feedback and increasing self-awareness can help encourage continuous learning. Consider barriers to learning from mistakes for new residents.
6	Interpreting Data for Informed Self-Assessment (Workshop)	Practice using the Transition to Residency Navigator technology application to schedule meetings with coaches, review and respond to goals, view learning portfolio items and interact with coaches.
7	Theories of Change: Helping Learners Achieve Their Goals (Workshop)	Discuss barriers to learning from feedback. Practice using a worksheet informed by the theory of immunity to change to identify barriers to growth and transformation. Practice using Eisenhower Decision Matrix to support time-management and organization.
8	Cultivating Resilience Through Adversity (Workshop)	Describe how resilience develops from engaging with adversity, and issues new residents face including imposter syndrome. Identify risk factors and protective factors for burnout and mental health problems. Articulate strategies to support recovery from work-related stress and achieve work-life integration.
9	Coaching Diverse Learners (Workshop)	Identify how bias creates obstacles for learners based on gender, race, ethnicity, learning styles and other factors. Using the Implicit Association Test and Circle of Trust Exercise, increase self-awareness around bias 9 and consider how bias may influence the coaching relationship.
10	It Takes a Village: Optimizing the Transition from the Trainee Perspective (Discussion with Residents)	Describe how interactions outside of the coaching relationship play a role in professional and personal development. Consider how to support socialization of medical students transitioning to residency.
n/a	Coaching Community of Practice (Ongoing Monthly Meetings)	Review practical questions related to coaching meetings and discuss individual coaching challenges with peers.
